# Identifying a gene expression signature of cluster headache in blood

**DOI:** 10.1038/srep40218

**Published:** 2017-01-11

**Authors:** Else Eising, Nadine Pelzer, Lisanne S. Vijfhuizen, Boukje de Vries, Michel D. Ferrari, Peter A. C. ‘t Hoen, Gisela M. Terwindt, Arn M. J. M. van den Maagdenberg

**Affiliations:** 1 Department of Human Genetics, Leiden University Medical Centre, Leiden, The Netherlands; 2 Department of Neurology, Leiden University Medical Centre, Leiden, The Netherlands

## Abstract

Cluster headache is a relatively rare headache disorder, typically characterized by multiple daily, short-lasting attacks of excruciating, unilateral (peri-)orbital or temporal pain associated with autonomic symptoms and restlessness. To better understand the pathophysiology of cluster headache, we used RNA sequencing to identify differentially expressed genes and pathways in whole blood of patients with episodic (*n* = 19) or chronic (*n* = 20) cluster headache in comparison with headache-free controls (*n* = 20). Gene expression data were analysed by gene and by module of co-expressed genes with particular attention to previously implicated disease pathways including hypocretin dysregulation. Only moderate gene expression differences were identified and no associations were found with previously reported pathogenic mechanisms. At the level of functional gene sets, associations were observed for genes involved in several brain-related mechanisms such as GABA receptor function and voltage-gated channels. In addition, genes and modules of co-expressed genes showed a role for intracellular signalling cascades, mitochondria and inflammation. Although larger study samples may be required to identify the full range of involved pathways, these results indicate a role for mitochondria, intracellular signalling and inflammation in cluster headache.

Cluster headache is a relatively rare brain disorder, typically characterized by multiple-daily, short-lasting (15–180 min) attacks of excruciating, unilateral, (peri-)orbital or temporal headache associated with ipsilateral facial autonomic symptoms and restlessness^1^,^2^. In up to 90% of patients, attacks strike in defined periods of several weeks to months alternating with attack-free periods of several months to years (episodic cluster headache). In the remaining 10%, attacks keep on recurring without attack-free periods longer than a month (chronic cluster headache). The lifetime prevalence is 0.05–0.4%^3^,^4^ and the male-to-female ratio 4.4: 1^4^,^5^.

The etiology of cluster headache is unknown. Triggered by neuroimaging data and the circadian and annual rhythmicity of attacks and periods of cluster headache, an important role has been postulated for the hypothalamus and the sleep-, pain-, and autonomic function-modulating, hypothalamic neuropeptide hypocretin (orexin)^6^,^7^,^8^,^9^. Activation of the trigeminovascular system and possibly associated inflammatory processes have been implicated in causing and aggravating the pain^10^,^11^,^12^. Although family members of cluster headache patients seem to be at increased risk of developing cluster headache^3^, indicating contribution of genetic factors, genetic studies into cluster headache have been largely unsuccessful^13^,^14^. Some studies suggested involvement of the *HCRTR2* gene that encodes the hypocretin type 2 receptor, but this finding could not be confirmed in other studies^15^. A main complicating factor for genetic association studies in cluster headache is that they included at best only a few hundred cases rather than the many thousands which are usually required for such studies.

Gene expression profiling is an alternative approach to identify disease genes and pathways requiring only several tens of cases. However, as well-characterized human post-mortem brain samples are difficult to obtain in these numbers, gene expression profiling studies for brain disorders have been mostly performed in whole blood samples. Promising results were obtained for epilepsy^16^ and psychiatric^17^,^18^ and neurodegenerative disorders^19^,^20^,^21^,^22^,^23^, with gene expression differences similar to those observed in post-mortem brain material^19^,^20^,^21^,^22^,^23^. Two gene expression profiling studies using microarray technology have been performed in cluster headache^24^,^25^. In one, whole blood gene expression levels from three cluster headache patients and three controls were compared; 90 differentially expressed genes were identified, including upregulated calcium-binding proteins suggesting a possible role for non-infectious inflammation^25^. In the other study, immortalized lymphoblastoid cell lines from eight lithium-responsive cluster headache patients and 10 controls were compared^26^. Over 1,100 differentially expressed genes were identified of which many are involved in endoplasmic reticulum protein processing^25^. Remarkably, only 10 genes overlapped between both studies, of which six showed differential expression when comparing the same patient outside and during a cluster period in-between attacks^24^. The limited overlap between these studies is likely explained by the small sample sizes and differences in gender, age, medication, tissues, and other factors.

RNA-sequencing (RNA-seq) is a deep sequencing-based technique that is more robust and detects a wider range of transcripts than microarray technology^26^. In the present study, we used RNA-seq to compare whole blood gene expression profiles of 39 well characterized participants with cluster headache (19 episodic; 20 chronic) and 20 matched controls. Besides analysing the RNA-seq data per gene, a clustering approach was applied to group genes into modules based on co-expression and study the association of these modules with cluster headache. Special attention was given to hypocretin-related genes and genes previously found to be differentially expressed in cluster headache.

## Results

Whole blood gene expression profiles of 40 participants with cluster headache (20 episodic; 20 chronic) and 20 controls were obtained using an RNA-seq approach. The RNA-seq data of one participant with episodic cluster headache were excluded because of inferior RNA-seq data quality (Table 1). In participants with cluster headache, blood samples were collected outside attacks; in 25/39 participants within one day from the last attack (10 episodic and 15 chronic patients), in 8/39 participants between one to seven days (4 episodic and 4 chronic patients), and in the remaining 6 within 94 days (5 episodic and 1 chronic patient). Participants with episodic cluster headache were studied within a “cluster period” (i.e. preventative treatment is still necessary because when lowering the treatment the patient perceives upcoming, or first symptomatology, of attacks). There were no differences between participants with episodic and chronic cluster headache for attack frequency and medication use in the month prior to sampling. Leukocyte counts did not differ between participants with cluster headache and controls (Supplementary Table 1). On average 23.9 million high-quality paired-end sequencing reads were obtained per sample (range: 21.4–30.2 million). On average 91% (range: 88–93%) of reads could be uniquely aligned to the genome, of which 77% (range: 71–82%) to known exons. A total of 13,416 genes with sufficiently high expression (after removal of genes with<1 count per million (CPM) in a minimum of 15 samples) were present in our dataset.

### Identification of differentially expressed genes

**Figure 1:**
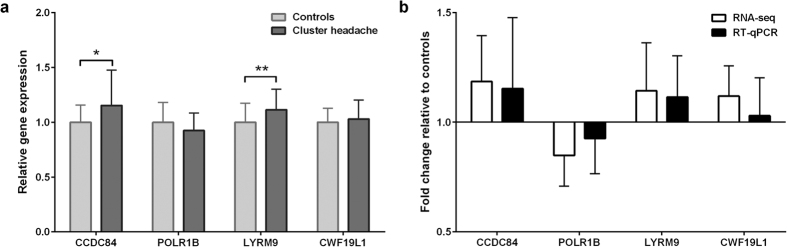
RT-qPCR validation of genes differentially expressed between cluster headache patients and controls. Four genes were chosen from the top 15 differentially expressed genes between participants with cluster headache and controls. Data were normalized to *TBP* mRNA expression. (**a**) Gene expression measured by RT-qPCR in the cluster headache group relative to the expression in the control group. (**b**) Comparison of fold-change of gene expression in participants with cluster headache relative to controls measured by RNA-seq and RT-qPCR. Means ± SD, *P < 0.05; **P < 0.01.

Expression differences between participants with cluster headache and controls were very modest, in fact no gene was found significant with a False Discovery Rate (FDR) of 0.05 or 0.1 (Table 2). Removing samples (n = 2) with a large time (>30 days) between inclusion and last attack did not affect results (Fig. S1). The Global Test, however, showed that the overall gene expression profile did differ between participants with cluster headache and controls (*P* = 0.037). This indicates that differential expression even after correction for multiple testing might have been identified if a larger sample size had been used. More lenient *P*-value thresholds were used for the selection of genes for follow up analyses (Table 2). Similar results were obtained for episodic and chronic cluster headache when these subsets were tested against controls (Table 2). RT-qPCR analysis of four genes from the top 15 differentially expressed genes identified in the RNA-seq dataset could not fully confirm differential expression, with only two genes (*LYRM9* and *CCDC84*) showing significantly differential expression (Fig. 1a). Irrespective of the statistical significance, we could replicate the *fold-changes* in expression (Fig. 1b).

### Brain, oxidation and intracellular signalling functions associated with cluster headache

As interpreting individual genes is highly susceptible to false-positive findings, several analysis methods were applied to explore the RNA-seq dataset at the level of functional gene sets. First, a pathway enrichment analysis was performed to identify functions overrepresented in lists of differentially expressed genes. Two *P*-value thresholds (*P* < 0.05 and *P* < 0.01; see Table 1 for the respective number of genes) for differential expression were used, and pathways that were overrepresented in both lists of genes were considered associated with cluster headache (Table 3). This way, the effect of possible false-positive associations was reduced. The top overrepresented pathway in genes with *P* < 0.05 is “GABA Receptor Signalling”. Most other overrepresented pathways involved signalling molecules, e.g. NUR77, CD28, Prolactin and IL-4.

Next, the Global Test was applied, which takes into account the full list of results, instead of merely a selection of differentially expressed genes. The Global Test is therefore especially suited to identify gene sets of which many genes show subtle associations while each individual gene association might be too small to be identified in the differential gene expression analysis^27^. Several functions associated with cluster headache are related to the brain, including “Calcium-release channel activity” (*P* = 0.0083), “Ganglioside metabolic process” (*P* = 0.0025) and Reactome pathways “GABA receptor activation” (*P* = 0.0066) and “Inhibition of voltage gated Ca^2+^channels via Gbeta/gamma subunits” (*P* = 0.0074) (Table 4). Other functions associated with cluster headache include several developmental and metabolism-related processes. Lastly, molecular function “Glutathione peroxidase activity” (*P* = 0.0013) and biological process “Response to hydroperoxide” (*P* = 0.006) are related to oxidation processes.

### Association with previously identified differentially expressed genes and hypocretin

The Global Test was also applied to study the overlap with genes previously identified as differentially expressed in cluster headache^24^,^25^. To this end, the 90^24^ and 1,172^25^ previously identified genes were imported as custom gene sets into the Global Test. No association was found with cluster headache with either gene set in our data set (*P* = 0.33 and *P* = 0.14, respectively). To study the association with cluster headache with hypocretin in the RNA-seq data, two custom gene sets were constructed in the STRING database and in the Euretos Knowledge Platform BRAIN. In STRING, 138 predicted functional partners of *HCRT* were identified, of which 49 were identified in blood with high enough expression. In BRAIN, 127 genes linked to *HCRT* were identified, of which 66 were identified in blood with high enough expression. No association was found with cluster headache for the custom hypocretin gene sets in the RNA-seq data (*P* = 0.39 and *P* = 0.19, respectively).

### Inflammation, mitochondria and intracellular signalling-related modules associated with cluster headache

**Figure 2:**
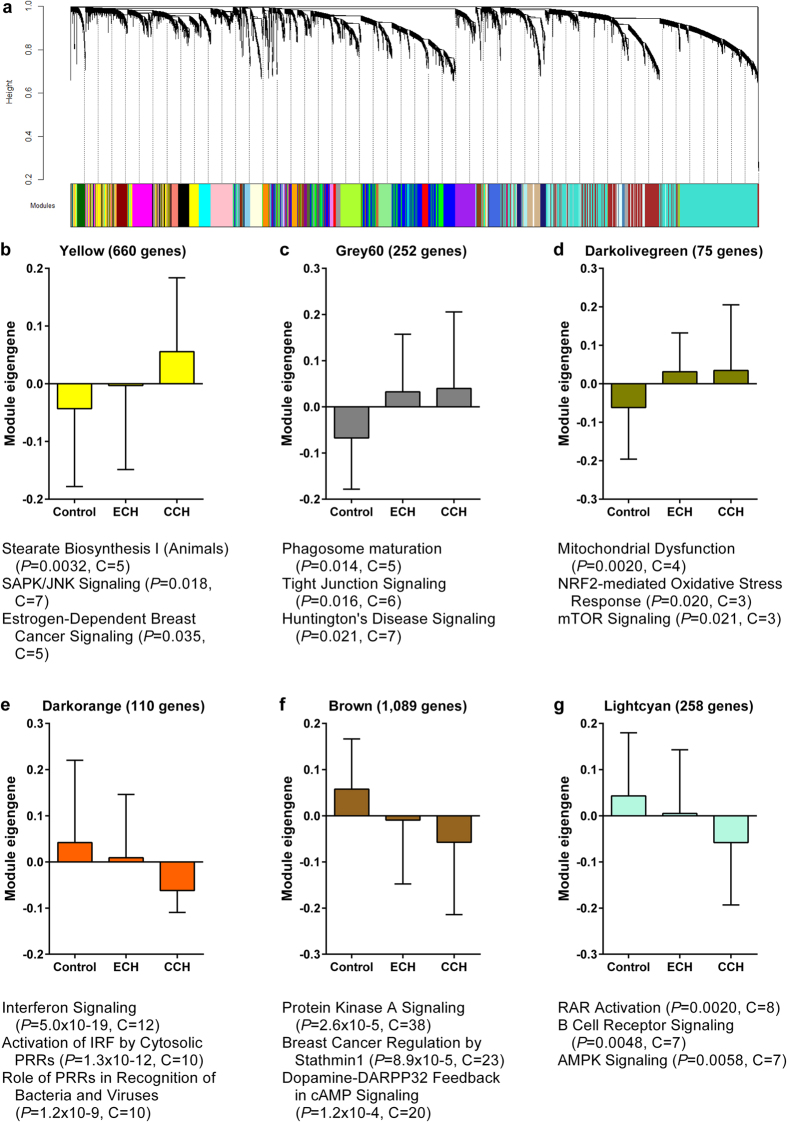
Gene co-expression modules in whole blood associated with cluster headache. Detection of co-expression modules in RNA-seq data of whole blood samples of cluster headache patients and controls using weighted gene co-expression network analysis (WGCNA). **(a)** The cluster dendrogram that gave rise to 40 modules, indicated by different colours. (**b**–**g**) Bar graphs of the module eigengenes in controls, episodic (ECH) and chronic cluster headache (CCH) of the six modules associated with cluster headache. The module eigengene can be considered the optimal summary of the module-specific gene expression pattern. Top three pathways significantly overrepresented in each module are listed below each bar graph. *P* = *P*-value, C = count of genes in the module belonging to the pathway.

Besides studying differentially expressed genes, weighted gene co-expression network analysis (WGCNA) was used to cluster genes in modules based on co-expression in the RNA-seq dataset, and to study the association of each of the modules with cluster headache. Because co-expression often results from genes being part of the same biological process, this method not only reduces the effect of multiple testing, but also greatly enhances the possibility for biological interpretation of an association. A total of 40 co-expression modules were identified, ranging in size from 34 to 2,903 genes, of which 6 showed an association with cluster headache (Fig. 2). Three modules contained genes with higher expression in cluster headache (i.e. “Yellow”, “Grey60” and “Darkolivegreen” modules), as visualized in the bar graphs of the module eigengenes, which can be considered as the optimal summary of the expression pattern of the genes in the modules (Fig. 2). These modules are enriched for genes involved in metabolic pathways and intracellular signalling (Yellow), phagocytosis and brain-related signalling (Grey60) and mitochondria (Darkolivegreen). Lower expression in cluster headache was seen for the modules “Darkorange”, “Brown” and “Lightcyan”. These modules contain genes involved in inflammation (Darkorange) and intracellular signalling (Brown and Lightcyan).

## Discussion

In this study we determined whole blood gene expression profiles of 19 participants with episodic cluster headache, 20 with chronic cluster headache, and 20 controls using an RNA-seq approach. To identify genes and pathways associated with cluster headache, analysis methods were applied that searched for association with single genes or for association with modules of co-expressed genes. Differential gene expression did not reveal a single cluster headache-associated gene that survived FDR multiple testing correction, hence differences in expression of genes in cluster headache, at best, are very modest.

As cluster headache-associated genes might be involved in multiple pathways, we conducted two types of functional enrichment analysis: functional enrichment in the lists of differentially expressed genes using a standard pathway enrichment method and the Global Test. These analyses suggest that inter- and intracellular signalling pathways involving brain-related molecules (e.g. GABA and ion channels) and inflammation-related molecules (e.g. CD28 and interleukin-4 (IL-4)), metabolism and oxidation might be involved in cluster headache. Although these findings should be interpreted with caution as inflammation-related processes seem to show up rather frequently in gene expression studies in blood^28^,^29^, inflammatory mediators such as IL-2 and soluble adhesion molecules have repeatedly been associated with cluster headache^11^,^12^,^30^,^31^. Abnormal expression of inflammatory genes in cluster headache was also observed in one of the earlier gene expression profiling studies^25^, although the genes identified in that study did not overlap with the genes found in our gene expression analysis.

Clustering analysis with WGCNA identified 40 modules of co-expressed genes in the gene expression data, of which six were associated with cluster headache. Associating gene co-expression modules to a disorder may provide potentially useful biological information with a disorder^32^, as gene co-expression modules capture a wide variety of biological factors, e.g. tissue composition and activity of transcription factors. Genes involved in inflammation and intracellular signalling were enriched in cluster headache-associated modules and the differential gene expression analysis, supporting a pathophysiological role of these processes in cluster headache. Association with mitochondria was only found in the co-expression analysis. This finding, however, might still be relevant as altered mitochondrial function has been detected with phosphorus magnetic resonance spectroscopy in skeletal muscle and brain in cluster headache^33^,^34^ and migraine (reviewed by Sparaco *et al*.^35^).

A role for hypocretin in the pathophysiology of cluster headache has previously been suggested^36^,^37^,^38^. Global Test analysis of the custom hypocretin gene sets, however, failed to reveal evidence for involvement of hypocretin in our RNA-seq data, which is in line with the absence of genetic association of *HCRTR2* gene variants with cluster headache in the largest sample studied so far^15^. Blood may, however, not be the most appropriate tissue to study possible deregulation of the hypocretin pathway. Only 49/139 and 66/127 genes of the two custom hypocretin gene sets could be measured reliably in blood. Moreover, changes in the hypocretin pathway may not be readily reflected at the RNA level and the timing of blood sampling in relation to cluster headache attacks might be critical. A recent study in cerebrospinal fluid, in which significantly reduced hypocretin peptide levels were found in cluster headache patients^39^, further indicates that analysis of other tissues than blood is required to identify all genes and pathways involved in cluster headache. Then again, the worth of blood is supported by the finding that several biomarkers, including PACAP-38^40^ and methionine-enkephalin^41^, show altered levels during the course of a cluster headache attack in blood plasma.

Our results are in sharp contrast with an earlier gene profiling study in which microarray of immortalized lymphoblastoid cell lines identified over 1,100 differentially expressed genes, suggesting rather massive gene deregulation in cluster headache^25^. We are unsure how to explain these marked differences. Possible reasons might be the small sample size of only eight patients who were also using lithium, and the differences in studied tissue. Both tissue type and medication use have previously been shown to have substantial effect on gene expression^42^,^43^,^44^. The Global Test analysis in our study did not identify associations for this and another gene set previously found differentially expressed^24^,^25^. Moreover, when comparing our top differentially expressed genes (*P* < 0.005) we could confirm only two genes that were identified in both previous studies: mastermind like transcriptional coactivator 2 (*MAML2*), a transcriptional coactivator involved in Notch signalling^45^, and lysozyme (*LYZ*), an antibacterial agent and possible inflammatory biomarker for Alzheimer disease^46^ and Niemann-Pick Type C^47^. Our results suggest that uncovering significant differentially expressed genes in cluster headache requires even larger study samples than the 59 we tested. Future studies on cluster headache and other episodic disorders should consider careful matching of study designs with previous studies, to enable result comparison and meta-analysis and should take into account the time of blood withdrawal relative to the occurrence of attacks (i.e. for cluster headache inside or outside an attack period).

In contrast to the previously published gene expression studies for cluster headache, we included episodic as well as chronic cluster headache patients and patients using various types of treatment (so the effect of a specific treatment on the differential gene expression analysis is low). Whereas including episodic and chronic cluster headache patients allowed stratification of patients by cluster headache subtype, given the fact that gene expression differences were only moderate and stratifying patients into subgroups would reduce power, most analyses compared all cluster headache patients with controls. Although the majority of patients (25 out of 39) was included within one day from their last attack, also patients were included with a broader range in time during inclusion and last attack. No information was collected about the time between the inclusion and the start and end of the cluster period in the episodic patients, therefore the gene expression analyses could not be controlled for periodicity of the cluster headache attacks. Importantly, removing patients over 30 days from their last attack did not change the results of the differential gene expression analysis, reflecting the robustness of the results. By allowing a broader time range between inclusion and last attack, our results provide information not only on the gene expression profile related to having an attack *per se*, but also to having cluster headache and being in an attack cluster. On the other hand, the heterogeneity of the cohort may have suppressed effect sizes and the number of genes that reached the different *P*-value thresholds. Resampling the same patients during different stages of the disease or including patients only on the same day of a cluster headache attack may aid to unravel differential gene expression patterns directly caused by a cluster headache attack from those caused by other cluster headache-related pathways.

In summary, we performed the largest gene expression profiling study to date in whole blood samples of 39 participants with cluster headache and 20 controls. Gene expression differences between cluster headache and controls were modest at the gene level. However, when analysed at the functional gene set level, genes associated with cluster headache were enriched for intracellular signalling cascades involving brain- and inflammation-related genes. Similar functions as well as mitochondrial functions were enriched in the cluster headache-associated modules of co-expressed genes. Larger study samples will be required to identify the full range of cluster headache-associated genes and pathways.

## Methods

### Participants

Male and female cluster headache patients and headache-free controls between 18 and 65 years of age were recruited from our specialised headache out-patient clinic and as part of the Leiden University Cluster headache Analysis (LUCA) programme^48^. Diagnoses of episodic and chronic cluster headache were made according to ICHD-III criteria^1^. In a personal interview, information was collected on cluster headache history, medication use during the month before sampling, and active smoking and number of pack years (Table 1, Supplementary Table 3). Special effort was made to record the time of the last cluster headache attack prior to blood sampling. No inclusion criteria were formulated regarding time since last cluster headache attack, but as a result of including patients at an outpatient headache clinic most patients were in a “cluster period”. For each two participants with cluster headache one control was included that was matched for gender, age and smoking habits. Demographic features of the three experimental groups (controls, episodic cluster headache and chronic cluster headache) that were used for the RNA-seq analysis were compared using analysis of variance (ANOVA) for continuous variables and a chi-square test for categorical variables. Student’s *t*-test and Fisher’s exact test were used for comparisons between episodic and chronic cluster headache groups. All participants provided written informed consent and the study was approved by the medical ethics committee of the LUMC. All experiments were carried out in accordance with the relevant guidelines and regulations.

Peripheral venous blood samples were drawn at the LUMC between May 2014 and September 2015 into: 1) EDTA-containing Vacutainer tubes for leukocyte differential count, and 2) PAXgene Blood RNA Tubes (PreAnalytiX, Qiagen, Hilden, Germany) for RNA isolation. Leukocyte counts were obtained using standard leukocyte differential count within two hours after blood collection. PAXgene Blood RNA Tubes were incubated overnight at room temperature and subsequently stored at −20 °C.

### RNA isolation and sequencing

PAXgene Blood RNA tubes were thawed and incubated for two hours at room temperature before RNA isolation using the PAXgene Blood miRNA kit (PreAnalytiX). Globin mRNA was depleted using the GLOBINclear™ Kit (Ambion, Austin, TX, USA). RNA quality was assessed using the Agilent 2100 Bioanalyzer (Agilent, Foster City, CA, USA); all samples had a minimal RNA integrity number (RIN) of 7. RIN values of controls, episodic, and chronic cluster headache samples were 8.5 ± 0.3, 8.5 ± 0.3 and 8.3 ± 0.5, respectively (*P*-value controls *vs* episodic *vs* chronic cluster headache = 0.23, ANOVA). TruSeq RNA-Seq libraries were constructed with a circa 160 base pair (bp) insert size, followed by 90 bp paired-end RNA-sequencing on the Illumina Hiseq4000 by BGI Tech Solutions in Hong Kong (www.bgitechsolutions.com).

### Sequencing data processing

RNA-seq reads were processed and aligned using the Gentrap pipeline (version 0.3.1) of the Sequencing Analysis Support Core (SASC) of the LUMC (https://humgenprojects.lumc.nl/sasc/). In brief, adapter sequences were clipped from RNA-seq reads using Cutadapt (version 1.5)^49^ and low-quality bases were removed using Sickle (version 1.33)^50^. Next, sequencing reads were aligned to the human genome reference GRCh38 using TopHat (version 2.0.13)^51^, allowing only for unique alignments (max_multihits = 1, read-mismatches = 2, read-gap-length = 2 and mate-inner-dist = 160). Refseq transcript annotations were obtained from the UCSC Genome Browser (http://genome.ucsc.edu/index.html), and read fragments aligned to known exons were counted per gene using Htseq (version 0.6.1p1)^52^. All analyses were performed on the gene level.

Quality of the RNA-seq dataset was assessed using FastQC^53^. Sample quality was assessed by: 1) hierarchical clustering of Spearman correlations between samples, 2) a multidimensional scaling (MDS) plot to visualize sample-to-sample distances, and 3) box plots of count distribution using R (version 3.2.2) and the Bioconductor package Limma (version 3.14.15)^54^. Next, the RNA-seq data was pre-processed for differential gene expression analysis. First, the data was filtered for low-expressed genes by removing genes with less than 1 CPM in at least 15 samples. Differences in library sizes were then normalized using the trimmed mean of M-values (TMM) function in the Bioconductor EdgeR package (version 3.10.5)^55^. The data was converted to a logarithmic scale using the Limma voom transformation.

### Differential gene expression analysis

Differential gene expression between cluster headache or the episodic or chronic subsets and control samples was calculated in Limma by fitting a linear model. To this end, data were normalized for age, gender, current smoking status, and leukocyte counts (basophils, eosinophils, lymphocytes, monocytes and neutrophils). Effects of medication use on differential gene expression were visualized in a heatmap of the top differentially expressed genes. Samples did not cluster on medication use, therefore, medication was not included as a cofactor in the differential gene expression analysis. FDR was used for multiple testing correction. In addition, several nominal *P*-value thresholds were applied to classify genes as differentially expressed for functional annotation (Table 2). The Global Test package was applied to test the overall gene expression data for differences between cluster headache and controls^28^.

### Weighted gene co-expression network analysis

WGCNA was used to construct gene modules based on pairwise correlations between gene expression levels in the RNA-seq data^56^,^57^. First, outlier samples were removed more strictly than for the differential gene expression analysis, as they can have large impact on co-expression values. Seven additional samples were removed based on Spearman correlation analysis between samples and MDS plot inspection, leaving 18 control, 19 episodic and 15 chronic cluster headache samples. Next, a signed weighted adjacency matrix was calculated using the power of 15. The power of 15 was chosen from a range of 1 to 20, as it maximized the fit of the scale free topology (R^2^ > 0.8). Of the adjacency matrix, a topological overlap matrix-based dissimilarity measure was calculated that was used as input for hierarchical clustering. The dynamic tree-cutting algorithm was used to define the modules^58^. A total of 40 modules were identified, each with a minimal size of 30 genes. Pearson correlations between modules eigengenes (which can be considered the optimal summary of the module-specific gene expression pattern) and cluster headache were considered significant if *P* < 0.05.

### Functional annotation of gene sets and modules

Enrichment analysis of canonical pathways in the lists of differentially expressed genes and the cluster headache-associated modules was performed using Ingenuity Pathways Analysis (IPA, Ingenuity Systems^®^; http://www.ingenuity.com). Only experimentally observed links between genes and pathways were included. The full list of genes that remained after filtering of low-expressed genes (with less than 1 CPM in at least 15 samples) was used as reference gene set. Only pathways from which five or more genes were observed in a list of differentially expressed genes or in a module, or three or more in a module containing up to 100 genes, were included.

Next, the Global Test was applied to identify gene sets associated with cluster headache in the full list of genes^27^. The Global Test is especially designed to identify related genes consistently associated with a trait, but with small effects that may not reach significance when assessing individual genes. The Global Test might, therefore, identify more subtle functional associations with cluster headache than the gene set enrichment analysis in IPA. Gene sets were included if annotated by Gene Ontology (GO) terms or REACTOME pathways from the curated molecular signature database of the Broad institute (version 5.1) if they contained 10 or more genes. GO terms associated with cluster headache with *P* < 0.01 were summarized with REVIGO^59^ by removing redundant terms and only preserving GO terms with a maximum allowed similarity of 0.5.

Custom hypocretin gene sets were built manually in the STRING database for known and predicted protein associations (version 10, http://string-db.org/)^60^ and in the Euretos Knowledge Platform BRAIN (https://www.euretos-brain.com/). In STRING, a manual gene set was built from predicted functional partners of *HCRT* based on gene co-expression, high-throughput experiments, databases and text mining; only predicted functional partners with high confidence (confidence score of 0.9 or higher) were included. In BRAIN, all genes linked to the concepts “HCRT”, “HCRTC2” and “orexins” were included. These links are based on a wide variety of databases and text mining. Association of the custom hypocretin gene sets with cluster headache was calculated using the Global Test.

### RT-qPCR validation

Findings from the RNA-sequencing data analysis were validated in the same RNA samples by real-time quantitative PCR (RT-qPCR). To this end, first-strand cDNA was synthesized with the RevertAid H Minus First Strand cDNA synthesis kit using random hexamer primers (Thermo Scientific Fermentas, Vilnius, Lithuania). RT-qPCR experiments were carried out in duplicate on the CFX384 Real-Time PCR Detection System (Bio-Rad, Hercules, CA, USA) using iQ™ SYBR^®^ Green (Bio-Rad) as fluorophore and exon-spanning primers (Supplementary Table 2). *TBP* was selected as reference gene based on its low variability in the RNA-seq data and high stability in RT-qPCR analysis. The RT-qPCR data was analysed with Bio-Rad CFX Manager^TM^ Software (version 3.1). Statistical analysis was performed using a linear model, correcting for age, gender, current smoking status and leukocyte counts (basophils, eosinophils, lymphocytes, monocytes and neutrophils).

## Additional Information

**Accession codes:** Sequence data has been deposited at the European Genome-phenome Archive (EGA), which is hosted by the EBI and the CRG, under accession number EGAS00001001918.

**How to cite this article**: Eising, E. *et al*. Identifying a gene expression signature of cluster headache in blood. *Sci. Rep.* **7**, 40218; doi: 10.1038/srep40218 (2017).

**Publisher's note:** Springer Nature remains neutral with regard to jurisdictional claims in published maps and institutional affiliations.

## Supplementary Material

Supplementary Figure and Tables

## Figures and Tables

**Table 1:** Demographics of the experimental groups used in the study.

	Controls	Episodic CH	Chronic CH	*P*-value Controls *vs* CH	*P*-value Controls *vs* episodic *vs* chronic CH
Number of individuals	20	19	20		
Age (years)	42.2 ± 12.4	40.0 ± 9.0	45.0 ± 13.4	0.97^c^	0.39^c^
Gender (M/F)	15/5	15/4	15/5	1^d^	0.86^c^
Current smokers (n)	10	10	11	0.79^d^	0.90^d^
Packyears (n)	11.6 ± 15.4	8.4 ± 11.5	14.7 ± 17.3	0.99^c^	0.49^c^
Days since latest attack	—	6.8 ± 13.9	5.8 ± 20.8	—	0.70^e^
Attack frequency during month prior to sampling (#/day)	—	1.0 ± 1.3	1.9 ± 2.1	—	0.13^e^
Prophylactic treatment	—			—	
- Verapamil		8	10		0.76^f^
- Other^a^		4	10		0.096^f^
- None		7	3		0.29^f^
Acute treatment	—			—	
- Sumatriptan s.c.		13	15		0.73^f^
- Oxygen		6	11		0.22^f^
- Other^b^		3	4		1^f^
- None		2	3		1^f^

Table gives mean ± standard deviation or exact number. All prophylactic treatment listed is current treatment, and all acute treatment is used within one month before blood sampling. CH: cluster headache; F: females; M: males; RIN: RNA integrity value.

^a^Other prophylactics used include lithium, frovatriptan, prednisolone, pizotifen, amitriptyline, *mometasonfuroate* and propranolol.

^b^Other acute treatment used include morphine, naproxen, paracetamol, ibuprofen and rizatriptan. Medication is only included in the table when used by >10% of cluster headache patients.

^c^ANOVA.

^d^Chi-square test.

^e^Students *t*-test.

^f^Fisher exact test.

**Table 2:** Number of differentially expressed genes based on different *P*-value thresholds.

	*P* < 0.005	*P* < 0.01	*P* < 0.05	FDR < 0.05
CH *vs* controls	310	614	2,347	0
Episodic CH *vs* controls*	90	226	1,313	0
Chronic CH *vs* controls*	125	200	1,233	0

CH: cluster headache; FDR: false discovery rate.

^*^Post-hoc analysis for cluster headache subtypes of differentially expressed genes for cluster headache.

**Table 3:** Pathways significantly overrepresented in the genes differentially expressed in cluster headache.

Pathway	Threshold DEGs *P* < 0.05		Threshold DEGs *P* < 0.01	
	*P*-value	Count	*P*-value	Count
GABA Receptor Signaling	0.00053	13	0.043	4
Calcium-induced T Lymphocyte Apoptosis	0.0015	18	0.028	6
Nur77 Signaling in T Lymphocytes	0.0066	15	0.0040	7
CD28 Signaling in T Helper Cells	0.0098	28	0.021	10
Cdc42 Signaling	0.015	29	0.014	11
Role of BRCA1 in DNA Damage Response	0.017	21	0.023	8
IGF-1 Signaling	0.022	24	0.025	9
Actin Nucleation by ARP-WASP Complex	0.023	15	0.0083	7
Prolactin Signaling	0.026	19	0.041	7
IL-4 Signaling	0.026	19	0.041	7
PTEN Signaling	0.043	25	0.045	9
Telomerase Signaling	0.044	23	0.026	9

*P*-value thresholds for differential gene expression are *P* < 0.05 and *P* < 0.01. Pathways are included if overrepresented in lists of differentially expressed genes based on both *P*-value thresholds. DEGs: differentially expressed genes.

**Table 4:** Differentially regulated functional gene sets identified with the Global Test.

Term ID	Description	*P*-value	Count
*Molecular Function*			
GO:0004602	Glutathione peroxidase activity	0.0013	11
GO:0015278	Calcium-release channel activity	0.0083	11
GO:0043560	Insulin receptor substrate binding	0.0085	11
GO:0031490	Chromatin DNA binding	0.0100	49
*Biological process*			
GO:0009235	Cobalamin metabolic process	0.0014	13
GO:0001573	Ganglioside metabolic process	0.0025	16
GO:0045682	Regulation of epidermis development	0.0026	30
GO:0046033	AMP metabolic process	0.0037	13
GO:0019082	Viral protein processing	0.0044	10
GO:1901068	Guanosine-containing compound metabolic process	0.0047	24
GO:0033194	Response to hydroperoxide	0.0060	14
GO:0007220	Notch receptor processing	0.0061	18
GO:0035357	Peroxisome proliferator activated receptor signaling pathway	0.0066	12
GO:0008206	Bile acid metabolic process	0.0077	20
GO:0032515	Negative regulation of phosphoprotein phosphatase activity	0.0083	13
GO:0070987	Error-free translesion synthesis	0.0091	14
GO:0045123	Cellular extravasation	0.0097	29
*Reactome*			
R-HSA-1660661	Sphingolipid de novo biosynthesis	0.0022	24
R-HSA-428157	Sphingolipid metabolism	0.0043	48
R-HSA-977444	GABA B receptor activation	0.0064	18
R-HSA-977443	GABA receptor activation	0.0066	19
R-HSA-997272	Inhibition of voltage gated Ca^2+^channels via Gbeta/gamma subunits	0.0074	11
R-HSA-400042	Inhibition of insulin secretion by adrenaline noradrenaline	0.0075	16
R-HSA-1296065	Inwardly rectifying K channels	0.0089	12
