# Supplementary material for: Identifying a gene expression signature of cluster headache in blood
Source: Sci Rep. 2017 Jan 11;7:40218. doi: 10.1038/srep40218 (PMC5225606; doi:10.1038/srep40218)
Supplement: Supplementary Figure and Tables [file srep40218-s1.pdf]

Identifying a gene expression signature of cluster headache in blood

E. Eising[1], N. Pelzer[2], L.S. Vijfhuizen[1], B. de Vries[1], M.D. Ferrari[2], P.A.C. 't Hoen[1], G.M. Terwindt[2], A.M.J.M. van den Maagdenberg[1,2]*

[1]Department of Human Genetics, Leiden University Medical Centre, Leiden, The Netherlands

[2]Department of Neurology, Leiden University Medical Centre, Leiden, The Netherlands

Supplementary figures

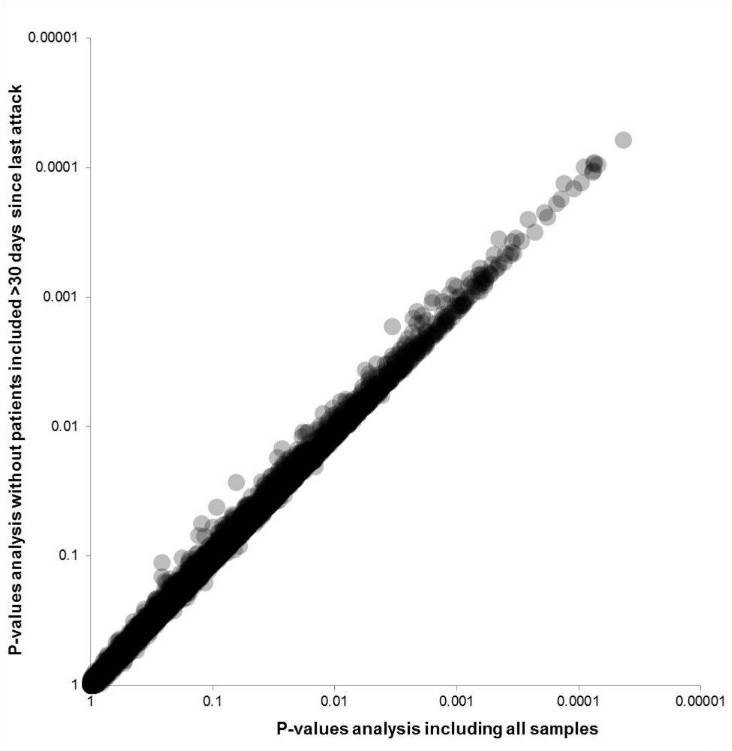

Supplementary Figure 1: Similar results were obtained for differential gene expression analyses with and without patients included over 30 days from their last attack. P-values are shown on logarithmic scale.

Supplementary tables

Supplementary Table 1: Leukocyte counts

**WBC**	**Controls**	**Episodic CH**	**Chronic CH**	***P*-value Controls *vs* CH[a]**	***P*-value Controls *vs* episodic *vs* chronic CH[b]**
Basophils (x 10^9/L)	0.024 ± 0.009	0.026 ± 0.016	0.033 ± 0.019	0.081	0.051
Eosinophils (x 10^9/L)	0.15 ± 0.08	0.13 ± 0.10	0.18 ± 0.11	0.78	0.37
Monocytes (x 10^9/L)	0.54 ± 0.20	0.56 ± 0.19	0.64 ± 0.17	0.29	0.11
Neutrophils (x 10^9/L)	4.26 ± 1.66	4.77 ± 1.71	5.04 ± 1.84	0.17	0.17
Lymphocytes (x 10^9/L)	2.22 ± 0.83	2.09 ± 0.82	2.56 ± 0.78	0.64	0.36

CH: cluster headache. [a]Students *t*-test, [b]ANOVA.

Supplementary Table 2: RT-qPCR primers

**Primer target**	**Forward sequence**	**Reverse sequence**	**PCR Product size (bp)**
CCDC84	5’-AACACCTGAGCCATGGAAAC-3’	5’-TGTTGGTTGCTTTCTTGTGC-3’	87
POLR1B	5’-AGCTGCGGAGTTCCTGTTTA-3’	5’-ATCAGGATTGTCCTCCATGC-3’	136
LYRM9	5’-GCATCCAGCAGCATTACAAG-3’	5’-GCTGGATTCTCTCAGGGTTG-3’	81
CWF19L1	5’-ACAGCCAGGAGCAGCATATT-3’	5’-CTCCCTTCCAAACTGCAAAG-3’	97
TBP	5’-TATAATCCCAAGCGGTTTGC-3’	5’-GCACACCATTTTCCCAGAAC-3’	90

Supplementary Table 3: Extended clinical and treatment information

Patient	Age (years)	Gender	CH subtype	Days since last attack	Attack frequency (#/day)	Active smoker	Pack years	Prophylactic treatment	Acute treatment	Other medication
1	43	F	ECH	0	1.3	0	0	-	Sumatriptan	-
2	44	M	ECH	0	0.6	1	29	-	Oxygen	-
3	27	F	ECH	0	0.9	0	0	Topiramate 2x 50 mg/day	Sumatriptan	Cetirizine
4	28	M	ECH	1	3.3	1	0	-	-	Quetiapine 25 mg/day
5	49	M	ECH	3	0.9	1	4	Verapamil 2x 120 mg/day	Sumatriptan	-
6	36	M	ECH	4	0.6	1	12	Verapamil 3x 120 mg/day	Sumatriptan, Oxygen, Paracetamol	Paracetamol
7	40	F	ECH	8	0.1	1	13	Verapamil 3x 240 mg/day	Sumatriptan, Oxygen	-
8	31	M	ECH	1	0.1	1	3	Verapamil 2x 240 mg/day	Sumatriptan	-
9	52	M	ECH	1	1.0	1	21	Verapamil 120 mg/day	Oxygen	Paracetamol
10	32	M	ECH	0	0.7	1	9	-	Sumatriptan, Morphine	-
11	52	M	ECH	2	0.9	0	0	-	Sumatriptan	-
12	52	M	ECH	1	2.0	1	38	Pizotifen 2x 1.5 mg/day, propranolol 80 mg/day	Sumatriptan, Oxygen	Omeprazole 40 mg/day, Ramipril 2.5 mg/day, Simvastatin 40 mg/day, Aspirin 80 mg/day
13	39	M	ECH	1	0.5	1	24	Verapamil 6x 80 mg/day	Sumatriptan	-
14	37	F	ECH	0	3.3	0	0	Verapamil 3x 120 mg/day	Sumatriptan	
15	31	M	ECH	60	0.0	0	0	-	-	-
16	51	M	ECH	18	0.1	0	5	Mometasone furoate 2x 50 mg/day	Sumatriptan, Ibuprofen	Loratadine 1 dose/dag, Desloratadine, Salbutamol
17	39	M	ECH	14	0.3	0	1	Verapamil 120 mg/day	Oxygen	Omeprazol 1 dose/day 40mg
18	30	M	ECH	11	0.6	0	1	-	Sumatriptan, Rizatriptan	-
19	29	M	ECH	4	0.1	0	0	Prednisolone 20 mg/day, Diclofenac 50 mg/day	Ibuprofen	
20	62	M	CCH	1	0.7	0	0	-	Sumatriptan	Pantoprazol
21	45	F	CCH	2	0.9	0	0	Topiramate 2x /dag	Frovatriptan, Sumatriptan, Oxygen	Propranolol 2x 40 mg/day, Ipratropium aerosol 1-4 doses/day, Pantoprazole 40 mg/day, Ciclesonide aerosol 1 dose/day, Levocetirizine 50 mg/day, Ibuprofen 2x

										600 mg/day.
22	26	M	CCH	0	1.3	1	2	Verapamil 7x 80 mg/day	Oxygen, Oxycodone	-
23	47	F	CCH	94	0.0	1	45	Pizotifen 1,5 mg/day	-	Fluticasone/formoterol
24	30	F	CCH	0	1.0	0	0	Verapamil 3x 240 mg/day	Oxygen	-
25	29	M	CCH	0	2.3	1	0	Lithium, 1200 mg/day	Sumatriptan	-
26	36	M	CCH	1	0.8	1	15	Lithium, 1400 mg/day	Sumatriptan, Oxygen	-
27	53	M	CCH	1	7.3	1	13	Verapamil, 9 x 80 mg/day	Sumatriptan	-
28	63	M	CCH	0	3.3	1	46	Topiramate 25 mg/day, Pizotifen 1,5 mg/day	-	Hydrochlorothiazide 25 mg/day, Diclofenac 100 mg/day, Salbutamol 800 mg/day, Metformin 500 mg/day, Clonazepam 2 mg/day, Lansoprazole 30 mg/day, Metamucil 3.4 mg/day, Atorvastatin 40 mg/day, Fluticasone propionate 1.6 mL/day, Amlodipine 10 mg/day
29	62	M	CCH	0	1.0	0	0	Verapamil 3x 240 mg/day, Frovatriptan 2x 2,5 mg/day	Sumatriptan	Carbasalate calcium 100 mg/day, Dipyridamole 200 mg/day, Omeprazole 20 mg/day
30	48	M	CCH	4	0.7	1	36	Verapamil 480 mg/day, Amitriptyline 25 mg/day	Sumatriptan, Oxygen	-
31	27	M	CCH	0	8.7	0	2	Topiramate 2x 50 mg/day	Sumatriptan, Oxygen	-
32	62	M	CCH	1	1.0	1	44	Verapamil 3x 240 mg/day	Sumatriptan	-
33	40	M	CCH	0	2.7	1	9	Verapamil, 480 mg/day	Sumatriptan	-
34	44	M	CCH	0	0.5	0	4	Verapamil, 3x 240/day	Sumatriptan, Oxygen	-
35	50	M	CCH	1	1.8	1	10	Verapamil 2x 360 mg/day	Sumatriptan, Oxygen	-
36	53	F	CCH	1	0.2	1	35	Lithium 2x 400 mg/day	Sumatriptan, Oxygen	Naproxen, Carbasalate calcium 100 mg/day, Pravastatin 40 mg/day, Omeprazole 40 mg/day, Dipyridamole 2x 200 mg/day
37	40	F	CCH	6	0.3	0	23	Verapamil 5x 120 mg/day	Sumatriptan, Oxygen	
38	23	M	CCH	3	2.7	0	0	-	-	-
39	62	M	CCH	0	1.9	0	9	-	Sumatriptan, Oxygen, Paracetamol, Excedrin	-

M: male, F: female, ECH: episodic cluster headache, CCH: chronic cluster headache
